# Parental and staff perspectives of NICU research procedures

**DOI:** 10.1186/s12887-016-0600-3

**Published:** 2016-05-10

**Authors:** Christina Freibott, Ursula Guillen, Amy Mackley, Robert Locke

**Affiliations:** Columbia University, New York, NY USA; Christiana Care Health System, Division of Neonatology, 4745 Ogletown-Stanton Roads, MAP-1 Suite 217, Newark, DE 19713 USA; Sidney Kimmel Medical College at Thomas Jefferson University, Philadelphia, PA USA

**Keywords:** Infants, Research regulatory, Research ethics, Parental perceptions

## Abstract

**Background:**

There are limited data on parental perception of infant participation in minimal risk and minor increase above minimal risk research focusing on the NICU population. The study objective was to assess parental and NICU staff perceptions concerning minimal risk and minor increase above minimal risk in the NICU setting.

**Methods:**

Parents of infants and NICU staff were presented with a combination of 4 infant scenarios and 5 hypothetical research procedures. These assessed participants’ willingness to allow their infant to participate in research and their attitude towards obligation to assist future children. Linear and hierarchal linear models analyzed the association and interaction effects on the likelihood to consent to research procedures.

**Results:**

Sixty parents and 30 NICU staff members were surveyed. Parents’ acceptability for each of the five research procedures ranged from 31 % to 83 %. Parent gender, age, race/ethnicity, insurance, education and history of previous child in the NICU were not associated with the likelihood to consent to the research procedures. Acceptability for each of the five research procedures among NICU staff ranged from 19 % to 98 %. There were no significant differences between NICU staff’s and parents’ responses for 4 of 5 research procedures. A minority of parents and nurses (38.3 % and 40 % respectively), compared to a majority of physicians (66.7 %), agreed or strongly agreed that parents have a responsibility to involve their children in low risk medical research in order to help future children, even if this would not help their own child. Lower agreement with obligation to help future children (*p* < 0.01) and higher education (*p* = 0.01) were associated with a decreased likelihood to consent to research procedures.

**Conclusion:**

In our study population, common NICU-related research procedures were considered appropriate and acceptable to a diverse group of NICU parents representing a wide range of race/ethnic and socioeconomic strata. Current regulations guiding informed consent for minimal and minor increase over minimal risk research in the NICU environment appear ethically consistent with a diverse group of parents and providers.

## Background

Neonates are considered a vulnerable research population. As such, they receive special consideration and protections under the US Code of Federal Regulations (CFR) 45 CFR 46, Subparts A, B, D [1]. Therefore, when conducting research in this patient population, investigators have to ask themselves: What is safe and appropriate for the infant? [2–15] Who makes the decision for an infant to participate, and what goes into that decision? [2, 4, 5, 11, 16–23] Additionally, one also needs to consider the societal benefit of the research in question. If medicine is restricted to only performing research that has potential for direct benefit (or no harm), the advancement of science may be limited to some unknown degree and future infants with similar conditions may receive harm because of the lack of scientific advance.

Unlike older children who can indicate their understanding of and willingness to participate in a research study, parents are most often the primary surrogate decision-makers for neonates. Surveys of parents of older infants and children assessing their willingness or reluctance to allow their child to participate in research yield similar themes: concerns of medical research and research related risk; desire to advance generalizable medical knowledge; and desire to advance knowledge specific to their own child’s disease [2, 4, 5, 14, 18, 24, 25]. These studies indicate that parental beliefs in the appropriateness of enrolling their children in minimal risk and minor-increase-over-minimal-risk research with or without direct benefit align with the federal regulations. Common research procedures and general societal benefit obligation focusing on the premature, late-term, and term newborn infant in the Neonatal Intensive Care Unit (NICU) population has received less investigation [26–28].

Parents of infants in the NICU are not the only members of the NICU community whose perspective should be taken into consideration. NICU staff members spend considerable periods of time, from days to months, with infants that require NICU care. NICU staff’s attitudes toward research in the NICU have not been previously assessed. This study aims to assess the parental and NICU staff perspectives on research in the premature, late-term and term newborn infant NICU population and on the legitimacy of exposure to minimal risk or minor-increase-above-minimal-risk exposure in the NICU setting.

## Methods

This study was conducted at a large tertiary care hospital in Newark Delaware. Institutional Review Board (Christiana Care Health System, Newark, DE, USA) approval was obtained prior to initiating the study. Informed consent was obtained from all participants prior to enrollment in the study. It is also standard policy at our institution that corporate Human Resources (HR) must approve all research involving hospital staff.

NICU parents were recruited from one of four groups matching the clinical scenarios presented: (1) Parent of infant with gestational age <31 weeks at birth; (2) Parent of infant gestational age 34-36 weeks at birth; (3) Parent of term infant in the NICU with mild transient illness and expected length of stay <7 days; and (4) Parent of healthy term neonate. A convenience sample of 15 parents per group was chosen. NICU parents were approached for participation when they presented to the NICU for a routine visit during the study period. Parents in the Mother/Baby Unit were approached for participation during their hospitalization following delivery. This study was limited to English-speaking parents who were at least 18 years of age. Enrollment continued until 15 parents returned completed surveys in each group.

NICU nurses and physicians (attending neonatologist and neonatology fellows) were recruited for enrollment. Surveys were distributed to all 12 neonatologists and 12 neonatology fellows at our institution. A convenience sample of nurses was approached for enrollment during their regularly scheduled daytime shift. The first 15 physicians and 15 NICU staff nurses who returned completed surveys were included in the study.

Parents and clinicians were presented with a questionnaire containing four different infant scenarios each with five hypothetical research procedures. The questionnaire is a modification of the one developed by Sachdeva and Morris that include parents of infants in the cardiac intensive care unit [2]. In that study, parents were presented with 6 possible procedures. Of these, we retained 4 procedures. Two procedures were excluded since they are not typical procedures in the NICU/newborn nursery setting. Instead, a common NICU procedure was added. Participants were asked: “would you give permission for the following research procedure on your child if your child was (one of four infant scenarios)”.

The four different infant scenarios were: (1) Early Preterm ≤ 31 weeks gestation, (2) Late Preterm 34-36 weeks gestation, (3) Term: In NICU with transient hypoglycemia, (4) Term: Healthy on Mother-Baby Unit. The five research procedures were: (1) Drawing 1 extra vial of blood for research purposes when other blood is being obtained for clinical purposes (would not require an extra blood draw); (2) drawing 1 extra vial of blood for research purposes only (would require an extra blood draw); (3) 2 echocardiograms (heart ultrasound)–these are not associated with any pain, but occasionally some infants can feel slight discomfort; (4) MRI brain without sedation; and (5) MRI brain with sedation. These five procedures remained constant in each scenario. Participants marked their hypothetical willingness to allow their child to participate in these research procedures depending on each infant scenario. The levels of willingness were: (1) Definitely yes; (2) Probably yes; (3) Probably no; and (4) Definitely no. To control for bias associated with question order, the scenarios were presented in random order. Research procedures were not pre-assigned a specific degree of risk, since there is variability in the perception of risk between individuals [4].

Participants were also presented with an additional question designed to assess their willingness to consent on the basis of one’s obligation to benefit others. They were asked to consider the statement: “Parents have a responsibility to involve their children in low risk medical research to help future children, even if it will not help their own children”. Participants were asked to mark their corresponding level of agreement: (1) Strongly Agree, (2) Agree, (3) Neither Agree nor Disagree, (4) Disagree, (5) Strongly Disagree. The questionnaire was reviewed by a group of clinicians (physicians and nurses) and personnel with limited medical knowledge for content and clarity.

Parents were also asked to complete a brief demographic survey about themselves, such as: age, gender, race, ethnicity, highest education completed, number of children, know a child in the NICU or with a chronic medical condition, and type of insurance. Parents also answered questions about their infant, such as: gestational age, birthweight, age (in days) and diagnosis. Clinicians were asked to report their years of experience. Due to concerns from HR about the ability to remain de-identified, no other demographic information was collected from the NICU staff.

Data were analyzed by parametric and non-parametric as appropriate for the data characteristics. Chi-square was used for unadjusted analysis of the Likert scales. Linear and hierarchal linear models were used to analyze the association and interaction effect of parental and infant demographics, differences between parents and providers, and obligation belief on the likelihood to consent to research procedures.

## Results

A total of 115 parents were approached for enrollment. Of these, 6 parents were discharged or transferred prior to returning the survey; 9 never returned the survey; 8 returned partially completed surveys and were excluded; and 1 parent declined participation. Parent and infant demographics are presented in Table 1. There were no significant differences between the parent groups with respect to age, gender, race/ethnicity, insurance, and education. A total of 44 clinicians were approached for enrollment. The first 15 physician and the first 15 NICU nurse surveys returned were included in the study. The mean experience for clinicians was 10.1 ± 9.8 years.

**Table 1:** Parent and infant demographics

Parent demographics	*n* (%)
Age, yrs (mean ± SD)	29 ± 6
Female	41 (68)
Race/Ethnicity	
White/Non-Hispanic	19 (32)
Black/Non-Hispanic	17 (29)
Hispanic	19 (32)
Other	4 (7)
Public insurance	19 (32)
Education	
Enrolled or completed 4-yr college	28 (46)
Previous child in the NICU	10 (16)
Infant birthweight, g (mean ± SD; median and interquartile range)	2605 ± 1139; 2835 (1233–3289)
Postnatal age at time parent enrollment, days (mean ± SD; median and interquartile range)	19 ± 36; 4 (2–15)

Willingness to consent to a research procedure for their child for the five research procedures stratified by the four parent groups (based upon infant status) and health provider is presented in Table 2. Parents’ willingness to allow their child to participate in a research procedure ranged from 31 % to 83 % (Table 3). There were no significant differences between the parent groups when each research procedure was analyzed individually or grouped together as a whole (‘any research’). There was no difference between parental responses whose infant matched a scenario to the non-matching scenario groups. Thus, responses from all four-parent groups were combined for further analysis.

**Table 2:** Parent and health care provider likelihood to approve research procedure stratified by procedure and infant status

Research procedure	Responder		Definitely Yes (%)	Probably Yes (%)	Probably No (%)	Definitely No (%)
“Drawing 1 extra vial of blood for research purposes when other blood is being obtained for clinical purposes. (Would not require an extra blood draw.)”	Parent by Infant Status	NICU ≤ 31 wks	50	22	15	13
		NICU 34-36 wks	45	38	3	13
		NICU Term with hypoglycemia	45	45	8	3
		Well-Baby Nursery/Mother-Baby Unit (37-42 weeks)	45	43	10	3
	Healthcare Provider		66	32	2	0
“Drawing 1 extra vial of blood for research purposes only. (Would require an extra blood draw.)”	Parent by Infant Status	NICU ≤ 31 wks	12	32	37	20
		NICU 34-36 wks	28	42	15	15
		NICU Term with hypoglycemia	18	33	33	15
		Well-Baby Nursery/Mother-Baby Unit (37-42 weeks)	32	33	33	15
	Healthcare Provider		22	41	28	10
“2 Echocardiograms (heart ultrasound). These are not associated with any pain, but occasionally some infants can feel slight discomfort”	Parent by Infant Status	NICU ≤ 31 wks	33	55	12	5
		NICU 34-36 wks	22	52	12	14
		NICU Term with hypoglycemia	18	56	25	0
		Well-Baby Nursery/Mother-Baby Unit (37-42 weeks)	30	43	23	3
	Healthcare Provider		50	48	2	0
“MRI brain without sedation”	Parent by Infant Status	NICU ≤ 31 wks	20	40	20	20
		NICU 34-36 wks	20	25	27	28
		NICU Term with hypoglycemia	7	27	40	20
		Well-Baby Nursery/Mother-Baby Unit (37-42 weeks)	12	22	42	23
	Healthcare Provider		28	38	24	11
“MRI brain with sedation”	Parent by Infant Status	NICU ≤ 31 wks	15	38	27	30
		NICU 34-36 wks	12	18	37	33
		NICU Term with hypoglycemia	5	21	20	54
		Well-Baby Nursery/Mother-Baby Unit (37-42 weeks)	5	20	39	36
	Healthcare Provider		3	16	48	33

**Table 3:** Parental definitely or likely to approve research procedure

Research procedure	Parents definitely or likely to approve
Drawing 1 extra vial of blood for research purposes when other blood is being obtained for clinical purposes. (Would not require an extra blood draw.)	83 %
Drawing 1 extra vial of blood for research purposes only. (Would require an extra blood draw.)	58 %
2 Echocardiograms (heart ultrasound). These are not associated with any pain, but occasionally some infants can feel slight discomfort	77 %
MRI brain without sedation	43 %
MRI brain with sedation	31 %

Parent type (mother/father), parent age, race/ethnicity, insurance, education, or having a prior history of a child in the NICU, were not associated with the likelihood to consent to any of the research procedures. There was a statistically significant decreased willingness to participate in a research procedure with increased education. There was a statistically significant increase in willingness to participate in a research procedure with increasing postnatal age. However, this explained only small portion of the variability of the response around its mean (adjusted r-square = 0.05; *p* ≤ 0.05). All of the parents with older (>15d) postnatal age infants had infants born at ≤ 31 weeks gestation.

Clinicians’ willingness to allow their infant to participate in a research procedure ranged from 19 % to 98 % (Table 2). There were no significant differences between the two clinician groups (nurses and physicians) for all five research procedures. When compared with parents, healthcare providers as a whole, were more likely to agree to the echocardiograms (*p* = 0.003). There were no significant differences between providers and parents for the other four procedures.

Willingness to engage in research to benefit others was measured by a five-point Likert scale (Table 4). A minority of parents (38.3 %) and nurses (40 %), in contrast to a majority of physicians (66.7 %), agreed or strongly agreed that parents have a responsibility to involve their children in low-risk medical research in order to help future children, even if this would not help their own child. However, this difference was not statistically significant (*p* = 0.343). Lower agreement with obligation beliefs (*p* < 0.01) and higher education (*p* = 0.012) were associated with a decreased likelihood to consent to any research procedure. There was not a significant interaction between these two variables (obligation score and parental education) and the likelihood to consent.

**Table 4:** Agreement with responsibility to participate in research to help future children

“Parents have a responsibility to involve their children in low risk medical research to help future children, even if it will not help their own children.”					
	Strongly agree	Agree	Neither agree or disagree	Disagree	Strongly disagree
Parents	13.3 %	25.0 %	28.9 %	23.3 %	4.4 %
Nurses	13.3 %	26.7 %	20.0 %	33.3 %	6.7 %
Physicians	6.7 %	60.0 %	6.7 %	26.7 %	0.0 %

## Discussion

In this study, we investigated whether parents would likely consent for their infant to participate in research and whether in their decision making process, they considered an obligation to help other children, even when there would be no direct benefit to their own child. This study is one of the first studies to focus on NICU parental acceptance of research procedures. Our sample of parents included a wide range of infant conditions commonly seen in the NICU. A comparative group of parents whose infants were not in the NICU as well as a group of clinicians made up of neonatal physicians and nurses were also surveyed.

Procedures queried in our study ranged from obtaining extra blood during an already planned blood draw to performing an MRI with and without sedation. These are considered low-risk procedures in the NICU. Since there is variability in the perception of risk between individuals, we did not pre-assign a specific degree of risk to the research procedures [4]. Although an individual healthcare provider may have a strong internal opinion of risk for a given procedure, there is no uniformity across healthcare providers. For example, Shah found there was high variability between IRB chairs concerning the perception of risk [3]. In that study, only 18 % of IRB chairs categorized a single blood draw as greater than minimal risk. However, 48 % of IRB chairs categorized an MRI without sedation as minimal risk, while the remainder categorized is as greater than minimal risk or remained undecided [3].

In this study, there was broad parental and clinician acceptability with wide variability for the 5 presented research procedures. In this study, there was consistency across all parent groups. There was no evidence that parental gender, age, race/ethnicity or insurance status were associated with a willingness to consent to the research procedures. Parents with a college education were less likely to consent to research procedures.

The range of acceptability of research procedures across the parent groups was similar to the attitudes demonstrated by NICU healthcare providers in our study sample. This is consistent with previously reported range of acceptability in other healthcare providers [3]. Existence of parental diversity should support, not dissuade, from the ethical appropriateness of allowing parents to make the final determination of infant participation in research. Healthcare providers and regulatory entities should exercise caution when engaging in overly paternalistic behaviors, less they infringe on the rights of parents as the first proxy and supplement themselves in that role without full justification based upon harm [29].

Excessive healthcare provider or institutional paternalistic behavior can improperly compromise parental autonomy and individual and societal beneficence [29]. Minimal and minor-increase above minimal-risk research are optional, “opt-in”, events. Healthcare providers and institutional regulatory entities are appropriate to prohibit research that unequivocally poses excessive risks to the infant compared to potential benefits (direct individual or societal). Research that is minimal risk or minor-increase-above-minimal-risk should be presented and discussed with parents through an appropriate informed consent process to permit the parent to decide. If 30 % of parents are comfortable with a procedure and 70 % of parents are not, limiting participation, when an “opt in” option can be given is provider-institutional paternalism (“secondary paternalism”) that unnecessarily and improperly compromises parental autonomy and choice, without providing additional protection to family units (parent-infant).

Similar to the parental and provider attitudes towards consent, there was variability in the strength in belief towards one’s obligation to consent to help future children. A feeling of obligation to help other children was associated with a willingness to consent to a research procedure. Further research into NICU parental and provider attitudes towards beneficence, societal justice and research deserve exploration.

These results provide practical supportive evidence that approving (or denying) research containing low-risk procedures may be acceptable to a meaningful proportion of NICU parents under conditions of informed consent. Given the wide variability in acceptability of different research procedures, informed consent gives parents control and allows them to exercise their status as the primary proxy decision-maker for their infant. As the events created by the fallout from the SUPPORT study demonstrate, there can be a misunderstanding of the risks of everyday clinical NICU care practices that supersede research to clarify those risks and benefits that are not fully understood by parents, media or regulators [30–32].

The purpose of this study was to enact a practical evaluation of whether the current commonly engaged ethical construct for research is acceptable to actual parents of NICU infants. A limitation of this study is its hypothetical nature. It is unclear if parents would make the same choices if presented with actual research studies rather than hypothetical scenarios. The study used a convenience sample of parents and as such may be biased by the selection of parents who presented to the NICU. Although there was a wide range of race/ethnicity and socioeconomic strata in the study group, bias may exist. The study sample was limited to English speaking parents only, though culturally inclusive of the Hispanic population (32 % of study parents). The study was not designed to specifically evaluate the interaction of race/ethnicity, culture, parental-infant characteristics and willingness to participate at an individual level, but whether participating in medical research that includes medical procedures that may carry minimal risk or minor increase over minimal risk is potentially acceptable to a diverse group of NICU parents. The study was also not designed to formulate a comprehensive list of procedures under specific conditions nor focus differences between parents and various health providers. A much larger sample size is needed to appreciate these potential differences.

## Conclusions

In our study population, there was variability in the acceptability of common NICU-related research procedures to a diverse group of NICU parents representing a wide range race/ethnic and socioeconomic strata. Regulations guiding informed consent for individual parent-determined approval/denial-of-participation in minimal and minor increase over minimal risk research in the NICU environment appear to be ethically consistent with the attitudes of parents and NICU providers towards common research procedures.

### Ethics approval and consent to participate

This study was approved by the IRB of Christiana Care Health System, Newark, DE, USA. Parental consent was obtained. Institutional IRB Study ID#: DDD602708.

### Consent for publication

Not applicable.

### Availability of data and materials

This study involves clinical personal subject data. Informed consent and IRB approval was obtained for public sharing and presentation of the data in aggregate anonymous form only. Please see manuscript and Tables 1, 2, 3 and 4 for additional information.

## Abbreviations

IRB: Institutional Review Board
MRI: Magnetic Resonance Imaging
NICU: Neonatal Intensive Care Unit

## References

[1] (OHRP). Federal Policy for the Protection of Human Subjects (‘Common Rule’). http://www.hhs.gov/ohrp/regulations-and-policy/regulations/45-cfr-46/index.html. Accessed 7 May 2016.

[2] Sachdeva T, Morris MC (2013). Higher-hazard, no benefit research involving children: parental perspectives. Pediatrics.

[3] Shah S, Whittle A, Wilfond B, Gensler G, Wendler D (2004). How do institutional review boards apply the federal risk and benefit standards for pediatric research?. JAMA.

[4] Wendler D, Abdoler E, Wiener L, Grady C (2012). Views of Adolescents and Parents on Pediatric Research Without the Potential for Clinical Benefit. Pediatrics.

[5] Wendler D, Jenkins T (2008). Children’s and their parents’ views on facing research risks for the benefit of others. Arch Pediatr Adolesc Med.

[6] Wendler D, Varma S (2006). Minimal risk in pediatric research. J Pediatr.

[7] Weijer C, Miller PB (2007). Refuting the net risks test: a response to Wendler and Miller’s “Assessing research risks systematically”. J Med Ethics.

[8] Hirshon JM, Krugman SD, Witting MD (2002). Variability in institutional review board assessment of minimal-risk research. Acad Emerg Med.

[9] Nelson RM (2007). Minimal Risk. Yet Again. J Pediatr.

[10] Resnik DB (2005). Eliminating the daily life risks standard from the definition of minimal risk. J Med Ethics.

[11] Varma S, Jenkins T, Wendler D (2008). How do children and parents make decisions about pediatric clinical research?. J Pediatr Hematol.

[12] Westra AE, Wit JM, Sukhai RN, De Beaufort ID (2011). How best to define the concept of minimal risk. J Pediatr.

[13] Wendler D (2012). A New Justification for Pediatric Research Without the Potential for Clinical Benefit. Am J Bioeth.

[14] Westra AE, Sukhai RN, Wit JM, de Beaufort ID, Cohen AF (2010). Acceptable risks and burdens for children in research without direct benefit: a systematic analysis of the decisions made by the Dutch Central Committee. J Med Ethics.

[15] Binik A, Weijer C (2014). Why the debate over minimal risk needs to be reconsidered. J Med Philos (United Kingdom).

[16] Tait AR, Zikmund-Fisher BJ, Fagerlin A, Voepel-Lewis T (2010). Effect of various risk/benefit trade-offs on parents’ understanding of a pediatric research study. Pediatrics.

[17] Tait AR, Voepel-Lewis T, Malviya S (2004). Factors that influence parents’ assessments of the risks and benefits of research involving their children. Pediatrics.

[18] Brody JL, Annett RD, Scherer DG, Perryman ML, Cofrin KMW (2005). Comparisons of adolescent and parent willingness to participate in minimal and above-minimal risk pediatric asthma research protocols. J Adolesc Heal.

[19] Ballard HO, Shook LA, Desai NS, Anand KJS (2004). Neonatal research and the validity of informed consent obtained in the perinatal period. J Perinatol.

[20] Caldwell PHY, Dans L, de Vries MC (2012). Standard 1: Consent and Recruitment. Pediatrics.

[21] Cameron MA, Marsillio LE, Cushman LF, Morris MC (2011). Parents’ perspectives on the consent approach for minimal- risk research involving children. IRB Ethics Hum Res.

[22] Fisher HR, McKevitt C, Boaz A (2011). Why do parents enrol their children in research: a narrative synthesis. J Med Ethics.

[23] Fogas BS, Oesterheld JR, Shader RI (2001). A retrospective study of children’s perceptions of participation as clinical research subjects in a minimal risk study. J Dev Behav Pediatr.

[24] Wendler D, Belsky L, Thompson KM, Emanuel EJ (2005). Quantifying the federal minimal risk standard: implications for pediatric research without a prospect of direct benefit. JAMA.

[25] Morris MC (2012). Pediatric Participation in Non-Therapeutic Research. J Law, Med Ethics.

[26] Singhal N, Oberle K, Burgess E, Huber-Okrainec J (2002). Parents’ perceptions of research with newborns. J Perinatol.

[27] Hoehn KS, Nathan A, White LE (2009). Parental perception of time and decision-making in neonatal research. J Perinatol.

[28] Ward FR (2009). Chaos, vulnerability and control: parental beliefs about neonatal clinical trials. J Perinatol.

[29] Miller RB. Children, Ethics and Modern Medicine. Indiana University Press; 2003.

[30] Lantos JD, Feudtner C (2015). SUPPORT and the Ethics of Study Implementation: *Lessons for Comparative Effectiveness Research from the Trial of Oxygen Therapy for Premature Babies*. Hastings Cent Rep.

[31] Drazen JM, Solomon CG, Morrissey S, Greene MF (2015). Support for SUPPORT. N Engl J Med.

[32] Drazen JM, Solomon CG, Greene MF (2013). Informed Consent and SUPPORT. N Engl J Med.

